# Crystal structure of *cis*-aqua­chlorido­bis­(1,10-phenanthroline-κ^2^ *N*,*N*′)chromium(III) tetra­chlorido­zincate monohydrate from synchrotron data

**DOI:** 10.1107/S2056989015003266

**Published:** 2015-02-21

**Authors:** Dohyun Moon, Jong-Ha Choi

**Affiliations:** a Pohang Accelerator Laboratory, POSTECH, Pohang 790-784, Republic of Korea; b Department of Chemistry, Andong National University, Andong 760-749, Republic of Korea

**Keywords:** crystal structure, synchrotron radiation, 1,10-phenanthroline, chloride ligand, aqua ligand, *cis*-geometry, chromium(III) complex

## Abstract

The structure of the title compound, [CrCl(C_12_H_8_N_2_)_2_(H_2_O)][ZnCl_4_]·H_2_O, has been determined from synchrotron data. The Cr^III^ ion is bonded to four N atoms from two 1,10-phenanthroline (phen) ligands, one water mol­ecule and a Cl atom in a *cis* arrangement, displaying an overall distorted octa­hedral coordination environment. The Cr—N(phen) bond lengths are in the range of 2.0495 (18) to 2.0831 (18) Å, while the Cr—Cl and Cr—(OH_2_) bond lengths are 2.2734 (7) and 1.9986 (17) Å, respectively. The tetra­hedral [ZnCl_4_]^2−^ anion is slightly distorted owing to its involvement in O—H⋯Cl hydrogen bonding with coordinating and non-coordinating water mol­ecules. The two types of water mol­ecules also inter­act through O—H⋯O hydrogen bonds. The observed hydrogen-bonding pattern leads to the formation of a three-dimensional network structure.

## Abstract 2

The Cr^III^ ion in the title complex is coordinated by two 1,10-phenanthroline (phen) ligands, one water mol­ecule and a chloride in a *cis* geometry, displaying a distorted octa­hedral environment. The [ZnCl_4_]^2−^ anion has a slightly distorted tetra­hedral coordination geometry.

## Chemical context   

Chromium(III) complexes with polypyridine ligands are particularly inter­esting because of their long lifetimes, thermal stabilities and tunable excited states. These complexes are promising materials for the development of new mol­ecule-based magnets, solar energy storage media or tunable solid state lasers (Powell, 1998 ▸; Dreiser *et al.*, 2012 ▸; Scarborough *et al.*, 2012 ▸). As a prerequisite for these applications, a detailed study of the structural and spectroscopic properties is needed. Therefore, we have been inter­ested in the preparation, crystal structures and spectroscopic properties of chromium(III) complexes containing mixed various ligands (Choi *et al.*, 2004**a* ▸,b* ▸, 2007 ▸; Choi, 2009 ▸; Choi & Lee, 2009 ▸; Choi & Moon 2014 ▸; Moon & Choi, 2015 ▸).
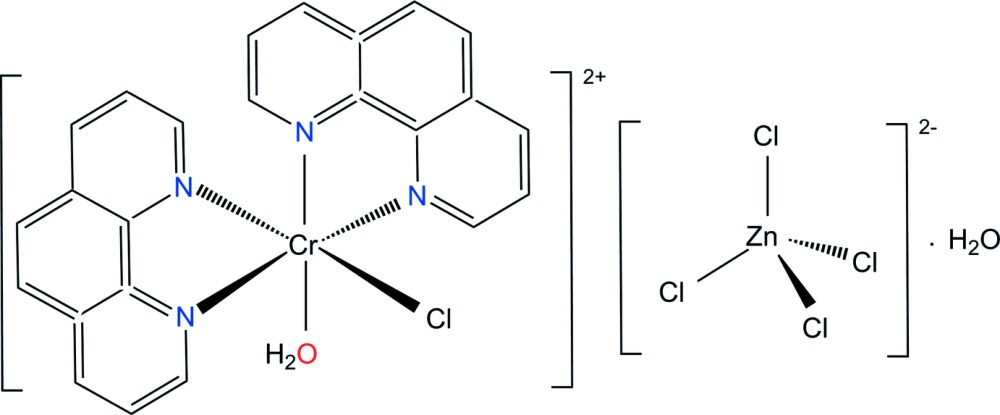


We report here on the synthesis and crystal structure of the title compound, [CrCl(phen)_2_(H_2_O)][ZnCl_4_]·H_2_O (phen = 1,10-phenanthroline), (I).

## Structural commentary   

**Figure 1:**
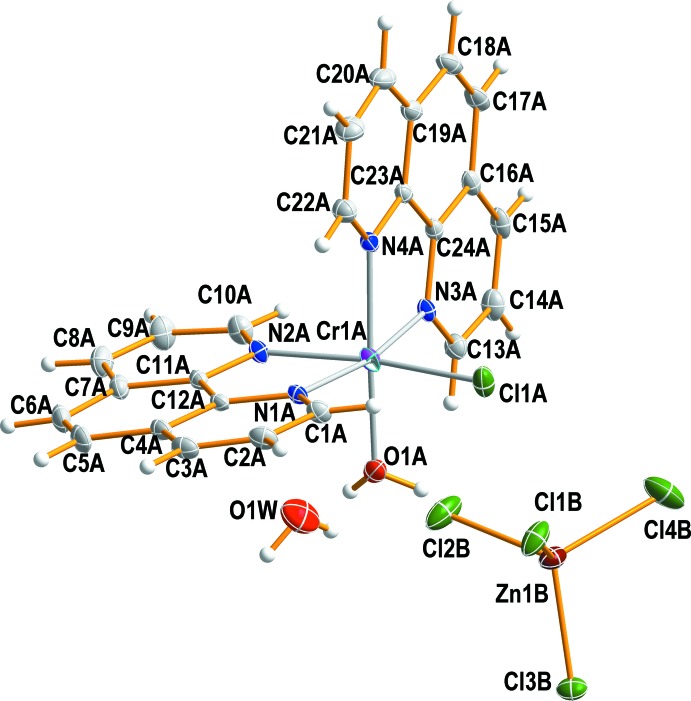
The structure of the mol­ecular components in (I), showing the atom-numbering scheme. Non-H atoms are shown as displacement ellipsoids at the 50% probability level.

In the mol­ecular structure of (I), there is one chlorine atom and one water mol­ecule coordinating to the Cr^III^ ion in a *cis* arrangement with an O1*A*—Cr1*A*—Cl1*A* bond angle of 89.79 (5)°. The other coordination sites are occupied by four nitro­gen atoms from two phen ligands, displaying an overall distorted octa­hedral coordination environment (Fig. 1 ▸).

The Cr—N(phen) bond lengths are in the range of 2.0495 (18) to 2.0831 (18) Å and are in good agreement with those observed in [Cr(phen)_3_](ClO_4_)_3_·H_2_O (Luck *et al.*, 2000 ▸), *cis*-[CrF_2_(phen)_2_]ClO_4_·H_2_O (Birk *et al.*, 2008 ▸) or *cis*-[CrCl_2_(phen)_2_]Cl (Gao, 2011 ▸). The Cr—Cl and Cr—(OH_2_) bond lengths in (I) are 2.2734 (7) and 1.9986 (17) Å, respectively. The Cr—(OH_2_) bond length is comparable to those of 1.947 (4), 1.9579 (10) and 1.996 (4) Å found in *cis*-[Cr(dpp)(phen)_2_(H_2_O)](NO_3_)_2_·H_2_O·CH_3_CN [Hdpp = (C_6_H_5_O)_2_·PO_2_H] (Ferreira *et al.*, 1998 ▸), *cis*-[CrF(bpy)_2_(H_2_O)](ClO_4_)_2_·2H_2_O (Birk & Bendix, 2010 ▸) and *trans*-[CrF(3,2,3-tet)(H_2_O)](ClO_4_)_2_·H_2_O (3,2,3-tet = 1,5,8,12-tetra­aza­undeca­ne) (Choi & Lee, 2008 ▸), respectively. The Cr—Cl bond length in (I) is somewhat shorter than those of 2.2941 (15) and 2.3253 (7) Å found in *cis*-[CrCl_2_(phen)_2_]Cl (Gao, 2011 ▸) or *trans*-[Cr(Me_2_tn)_2_Cl_2_]Cl (Me_2_tn = 2,2-di­methyl­propane-1,3-di­amine) (Choi *et al.*, 2007 ▸), respectively. The Cl1*A*—Cr1*A*—N2*A* and N1*A*—Cr1*A*—N3*A* angles in (I) are 171.72 (5) and 169.79 (7)°, respectively. The bite angles N1*A*—Cr1*A*—N2*A* and N3*A*—Cr1*A*—N4*A* are 79.76 (5) and 80.23 (7)°.

The [ZnCl_4_]^2−^ anion and the second water mol­ecule remain outside the coordination sphere. The Zn^II^ atom in the complex anion exhibits a slightly distorted tetra­hedral coordination sphere caused by the influence of hydrogen bonding on the Zn—Cl bond lengths and the Cl—Zn—Cl angles. The Zn—Cl bond lengths range from 2.2443 (7) to 2.2854 (7) Å and the Cl—Zn—Cl angles from 107.54 (4) to 111.57 (3)°.

## Supra­molecular features   

**Figure 2:**
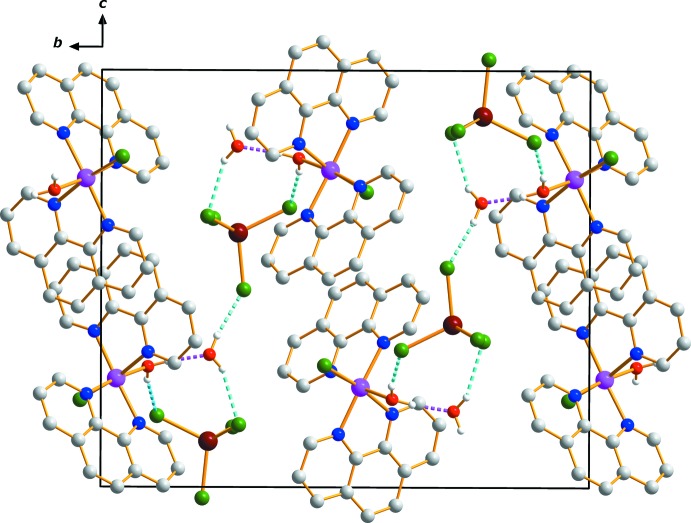
The crystal packing in (I), viewed along [100]. Dashed lines represent O—H⋯O (purple) and O—H⋯Cl (blue) hydrogen-bonding inter­actions.

The supra­molecular architecture involves hydrogen bonds including the O—H groups of coordinating and non-coord­inating water mol­ecules as donors, and the Cl atoms of the complex anion and the O atom of the solvent water mol­ecule as acceptors. Atom Cl3*B* of the [ZnCl_4_]^2−^ anion and the Cl1*A* ligand atom are not involved in hydrogen bonding. An extensive array of O—H—O and O—H⋯Cl contacts (Table 1 ▸) generates a three-dimensional network of mol­ecules stacked along the *a*-axis direction (Fig. 2 ▸). These hydrogen-bonded networks help to stabilize the crystal structure.

## Database survey   

A search of the Cambridge Structural Database (Version 5.35, May 2014 with one update; Groom & Allen, 2014 ▸) indicates a total of 36 hits for Cr^III^ complexes containing two bidentate 1,10-phenanthroline ligands. The crystal structures of *cis*-[Cr(dpp)(phen)_2_(H_2_O)](NO_3_)_2_·H_2_O·CH_3_CN (Ferreira *et al.*, 1998 ▸), [Cr(phen)_3_](ClO_4_)_3_·H_2_O (Luck *et al.*, 2000 ▸), *cis*-[CrF_2_(phen)_2_]ClO_4_ (Birk *et al.*, 2008 ▸) and *cis*-[CrCl_2_(phen)_2_]Cl (Gao, 2011 ▸) have been reported previously. However, no structures of complexes of [CrCl(phen)_2_(H_2_O)]^2+^ with any anions have been deposited.

## Synthesis and crystallization   

All chemicals were reagent-grade materials and used without further purification. The starting material, *cis*-[CrF_2_(phen)_2_]ClO_4_·H_2_O was prepared according to a literature procedure (Glerup *et al.*, 1970 ▸). Crude *cis*-[CrF_2_(phen)_2_]ClO_4_·H_2_O (0.2 g) was dissolved in 10 mL of 0.01 *M* HCl at 313 K, and 5 mL of 1 *M* HCl containing 1.2 g of solid ZnCl_2_ were added to this solution. The mixture was refluxed at 328 K for 30 min and then cooled to room temperature. The resulting solution was filtered and allowed to stand at room temperature for 3–5 days, giving purple crystals of (I) suitable for X-ray structural analysis.

## Refinement   

Crystal data, data collection and structure refinement details are summarized in Table 2 ▸. C-bound H atoms were placed in calculated positions (C—H = 0.95 Å) and were included in the refinement in a riding-model approximation with *U*_iso_(H) set to 1.2*U*_eq_(C). The H atoms of water mol­ecules (H1*O*1 and H2*O*1: H atoms of coordinating water; H1O*W* and H2O*W*: H atoms of solvent water) were located from difference Fourier maps and refined with restraints and an O—H distance of 0.84 (1) Å, with *U*_iso_(H) values of 1.2 *U*_eq_(O1*A*, O1*W*).

## Supplementary Material

Crystal structure: contains datablock(s) I. DOI: 10.1107/S2056989015003266/wm5123sup1.cif

Structure factors: contains datablock(s) I. DOI: 10.1107/S2056989015003266/wm5123Isup2.hkl

CCDC reference: 1049598

Additional supporting information:  crystallographic information; 3D view; checkCIF report

## Figures and Tables

**Table 1:** Hydrogen-bond geometry (, )

*D*H*A*	*D*H	H*A*	*D* *A*	*D*H*A*
O1*A*H1*O*1O1*W*	0.83(1)	1.72(1)	2.536(3)	170(3)
O1*A*H2*O*1Cl1*B*	0.84(1)	2.20(1)	3.0208(19)	168(3)
O1*W*H2*OW*Cl2*B*	0.86(1)	2.39(2)	3.172(3)	153(3)
O1*W*H1*OW*Cl4*B* ^i^	0.85(1)	2.31(1)	3.155(2)	170(3)

Symmetry code: (i) 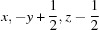.

**Table 2:** Experimental details

Crystal data	
Chemical formula	[CrCl(C_12_H_8_N_2_)_2_(H_2_O)][ZnCl_4_]H_2_O
*M*_r_	691.06
Crystal system, space group	Monoclinic, *P*2_1_/*c*
Temperature (K)	100
*a*, *b*, *c* ()	8.2710(17), 19.535(4), 16.934(3)
()	100.55(3)
*V* (^3^)	2689.8(10)
*Z*	4
Radiation type	Synchrotron, = 0.62998
(mm^1^)	1.30
Crystal size (mm)	0.10 0.08 0.05

Data collection	
Diffractometer	ADSC Q210 CCD area detector
Absorption correction	Empirical (using intensity measurements) (*HKL3000sm SCALEPACK*; Otwinowski Minor, 1997 ▸)
*T*_min_, *T*_max_	0.881, 0.938
No. of measured, independent and observed [*I* > 2(*I*)] reflections	25530, 7554, 7016
*R*_int_	0.045
(sin /)_max_ (^1^)	0.696

Refinement	
*R*[*F* ^2^ > 2(*F* ^2^)], *wR*(*F* ^2^), *S*	0.040, 0.107, 1.02
No. of reflections	7554
No. of parameters	348
No. of restraints	6
H-atom treatment	H atoms treated by a mixture of independent and constrained refinement
_max_, _min_ (e ^3^)	1.96, 0.87

Computer programs: *PAL ADSC Quantum-210 ADX* (Arvai Nielsen, 1983 ▸), *HKL3000sm* (Otwinowski Minor, 1997 ▸), *SHELXT2014/5* (Sheldrick, 2015*a* ▸), *SHELXL2014/7* (Sheldrick, 2015*b* ▸), *DIAMOND* (Putz Brandenburg, 2014 ▸) and *publCIF* (Westrip, 2010 ▸).

## supplementary crystallographic information

### Crystal data

**Table:**

[CrCl(C_12_H_8_N_2_)_2_(H_2_O)][ZnCl_4_]·H_2_O	*F*(000) = 1388
*M**_r_* = 691.06	*D*_x_ = 1.706 Mg m^−^^3^
Monoclinic, *P*2_1_/*c*	Synchrotron radiation, λ = 0.62998 Å
*a* = 8.2710 (17) Å	Cell parameters from 65318 reflections
*b* = 19.535 (4) Å	θ = 0.4–33.6°
*c* = 16.934 (3) Å	µ = 1.30 mm^−^^1^
β = 100.55 (3)°	*T* = 100 K
*V* = 2689.8 (10) Å^3^	Block, purple
*Z* = 4	0.10 × 0.08 × 0.05 mm

### Data collection

**Table:**

ADSC Q210 CCD area-detector diffractometer	7016 reflections with *I* > 2σ(*I*)
Radiation source: PLSII 2D bending magnet	*R*_int_ = 0.045
ω scan	θ_max_ = 26.0°, θ_min_ = 2.2°
Absorption correction: empirical (using intensity measurements) (*HKL3000sm *SCALEPACK**; Otwinowski & Minor, 1997)	*h* = −11→11
*T*_min_ = 0.881, *T*_max_ = 0.938	*k* = −27→27
25530 measured reflections	*l* = −23→23
7554 independent reflections	

### Refinement

**Table:**

Refinement on *F*^2^	6 restraints
Least-squares matrix: full	Hydrogen site location: mixed
*R*[*F*^2^ > 2σ(*F*^2^)] = 0.040	H atoms treated by a mixture of independent and constrained refinement
*wR*(*F*^2^) = 0.107	*w* = 1/[σ^2^(*F*_o_^2^) + (0.0492*P*)^2^ + 5.8256*P*] where *P* = (*F*_o_^2^ + 2*F*_c_^2^)/3
*S* = 1.02	(Δ/σ)_max_ = 0.001
7554 reflections	Δρ_max_ = 1.96 e Å^−^^3^
348 parameters	Δρ_min_ = −0.87 e Å^−^^3^

### Special details

**Table:**

Geometry. All e.s.d.'s (except the e.s.d. in the dihedral angle between two l.s. planes) are estimated using the full covariance matrix. The cell e.s.d.'s are taken into account individually in the estimation of e.s.d.'s in distances, angles and torsion angles; correlations between e.s.d.'s in cell parameters are only used when they are defined by crystal symmetry. An approximate (isotropic) treatment of cell e.s.d.'s is used for estimating e.s.d.'s involving l.s. planes.

### Fractional atomic coordinates and isotropic or equivalent isotropic displacement parameters (Å^2^)

**Table:**

	*x*	*y*	*z*	*U*_iso_*/*U*_eq_	
Cr1A	0.77359 (4)	0.46841 (2)	0.23994 (2)	0.01080 (10)	
Cl1A	0.60790 (7)	0.54226 (3)	0.29174 (3)	0.02194 (13)	
O1A	0.58493 (19)	0.40363 (9)	0.21135 (9)	0.0188 (3)	
H1O1	0.586 (4)	0.3627 (7)	0.1986 (18)	0.023*	
H2O1	0.517 (3)	0.4046 (14)	0.2425 (16)	0.023*	
N1A	0.7218 (2)	0.51346 (9)	0.12699 (10)	0.0116 (3)	
N2A	0.9036 (2)	0.40349 (9)	0.17655 (10)	0.0130 (3)	
N3A	0.8666 (2)	0.42150 (9)	0.34777 (10)	0.0127 (3)	
N4A	0.9743 (2)	0.52941 (9)	0.27805 (10)	0.0129 (3)	
C1A	0.6368 (3)	0.57069 (11)	0.10513 (12)	0.0160 (4)	
H1A	0.5964	0.5966	0.1448	0.019*	
C2A	0.6054 (3)	0.59364 (11)	0.02519 (13)	0.0183 (4)	
H2A	0.5463	0.6349	0.0115	0.022*	
C3A	0.6605 (3)	0.55611 (12)	−0.03309 (13)	0.0180 (4)	
H3A	0.6377	0.5708	−0.0875	0.022*	
C4A	0.7514 (2)	0.49553 (11)	−0.01165 (12)	0.0145 (4)	
C5A	0.8122 (3)	0.45241 (12)	−0.06790 (13)	0.0201 (4)	
H5A	0.7896	0.4637	−0.1234	0.024*	
C6A	0.9019 (3)	0.39541 (12)	−0.04308 (13)	0.0205 (4)	
H6A	0.9400	0.3673	−0.0816	0.025*	
C7A	0.9397 (3)	0.37714 (11)	0.04016 (13)	0.0163 (4)	
C8A	1.0386 (3)	0.32049 (12)	0.07004 (14)	0.0215 (4)	
H8A	1.0858	0.2922	0.0347	0.026*	
C9A	1.0656 (3)	0.30693 (12)	0.15110 (15)	0.0241 (5)	
H9A	1.1320	0.2691	0.1722	0.029*	
C10A	0.9944 (3)	0.34918 (11)	0.20257 (13)	0.0190 (4)	
H10A	1.0120	0.3385	0.2582	0.023*	
C11A	0.8769 (2)	0.41770 (10)	0.09595 (11)	0.0117 (3)	
C12A	0.7805 (2)	0.47693 (10)	0.06979 (11)	0.0115 (3)	
C13A	0.8073 (3)	0.36762 (11)	0.38182 (13)	0.0169 (4)	
H13A	0.7095	0.3464	0.3546	0.020*	
C14A	0.8845 (3)	0.34138 (12)	0.45623 (13)	0.0199 (4)	
H14A	0.8392	0.3029	0.4787	0.024*	
C15A	1.0265 (3)	0.37163 (12)	0.49676 (12)	0.0186 (4)	
H15A	1.0802	0.3540	0.5470	0.022*	
C16A	1.0910 (3)	0.42887 (11)	0.46279 (12)	0.0155 (4)	
C17A	1.2356 (3)	0.46545 (12)	0.49998 (13)	0.0209 (4)	
H17A	1.2952	0.4500	0.5502	0.025*	
C18A	1.2888 (3)	0.52137 (12)	0.46518 (14)	0.0209 (4)	
H18A	1.3837	0.5450	0.4918	0.025*	
C19A	1.2040 (2)	0.54558 (11)	0.38867 (13)	0.0164 (4)	
C20A	1.2508 (3)	0.60401 (12)	0.34946 (15)	0.0208 (4)	
H20A	1.3461	0.6291	0.3725	0.025*	
C21A	1.1571 (3)	0.62439 (12)	0.27744 (15)	0.0226 (4)	
H21A	1.1858	0.6643	0.2510	0.027*	
C22A	1.0188 (3)	0.58561 (11)	0.24359 (13)	0.0182 (4)	
H22A	0.9546	0.6002	0.1941	0.022*	
C23A	1.0641 (2)	0.50980 (10)	0.35036 (12)	0.0123 (3)	
C24A	1.0064 (2)	0.45161 (10)	0.38781 (11)	0.0125 (3)	
Zn1B	0.36987 (3)	0.28015 (2)	0.39019 (2)	0.01831 (10)	
Cl1B	0.35510 (7)	0.38494 (3)	0.32928 (4)	0.02700 (15)	
Cl2B	0.57258 (8)	0.21748 (3)	0.35055 (5)	0.03248 (17)	
Cl3B	0.12374 (6)	0.22928 (3)	0.35577 (3)	0.01971 (13)	
Cl4B	0.42194 (10)	0.29190 (4)	0.52663 (4)	0.03734 (18)	
O1W	0.6215 (3)	0.27775 (11)	0.18258 (15)	0.0383 (5)	
H1OW	0.575 (5)	0.2623 (19)	0.1371 (10)	0.046*	
H2OW	0.589 (5)	0.2513 (17)	0.2168 (16)	0.046*	

### Atomic displacement parameters (Å^2^)

**Table:**

	*U*^11^	*U*^22^	*U*^33^	*U*^12^	*U*^13^	*U*^23^
Cr1A	0.01239 (16)	0.01388 (16)	0.00571 (15)	0.00007 (10)	0.00052 (10)	0.00026 (10)
Cl1A	0.0241 (3)	0.0262 (3)	0.0161 (2)	0.0080 (2)	0.00524 (19)	−0.00103 (19)
O1A	0.0145 (7)	0.0292 (8)	0.0123 (7)	0.0010 (6)	0.0014 (5)	0.0026 (6)
N1A	0.0131 (7)	0.0132 (7)	0.0076 (7)	−0.0004 (6)	−0.0002 (6)	0.0008 (6)
N2A	0.0166 (8)	0.0130 (7)	0.0089 (7)	0.0009 (6)	0.0014 (6)	0.0011 (6)
N3A	0.0155 (7)	0.0147 (7)	0.0076 (7)	0.0011 (6)	0.0013 (6)	0.0009 (6)
N4A	0.0129 (7)	0.0147 (8)	0.0108 (7)	0.0005 (6)	0.0013 (6)	−0.0025 (6)
C1A	0.0161 (9)	0.0165 (9)	0.0142 (9)	0.0027 (7)	−0.0004 (7)	0.0009 (7)
C2A	0.0180 (9)	0.0179 (9)	0.0167 (9)	0.0011 (7)	−0.0028 (7)	0.0036 (7)
C3A	0.0184 (9)	0.0211 (10)	0.0126 (9)	−0.0025 (8)	−0.0019 (7)	0.0050 (7)
C4A	0.0155 (8)	0.0188 (9)	0.0088 (8)	−0.0034 (7)	0.0009 (7)	0.0011 (7)
C5A	0.0259 (10)	0.0262 (11)	0.0087 (8)	−0.0029 (8)	0.0042 (7)	−0.0006 (8)
C6A	0.0261 (11)	0.0238 (10)	0.0130 (9)	−0.0024 (8)	0.0074 (8)	−0.0047 (8)
C7A	0.0200 (9)	0.0153 (9)	0.0147 (9)	−0.0013 (7)	0.0061 (7)	−0.0035 (7)
C8A	0.0271 (11)	0.0168 (9)	0.0223 (10)	0.0032 (8)	0.0090 (9)	−0.0029 (8)
C9A	0.0310 (12)	0.0177 (10)	0.0240 (11)	0.0086 (9)	0.0062 (9)	0.0015 (8)
C10A	0.0245 (10)	0.0163 (9)	0.0156 (9)	0.0052 (8)	0.0021 (8)	0.0022 (7)
C11A	0.0143 (8)	0.0127 (8)	0.0082 (8)	−0.0029 (6)	0.0021 (6)	−0.0005 (6)
C12A	0.0116 (8)	0.0143 (8)	0.0080 (8)	−0.0028 (6)	0.0005 (6)	−0.0001 (6)
C13A	0.0198 (9)	0.0171 (9)	0.0141 (9)	−0.0004 (7)	0.0038 (7)	0.0018 (7)
C14A	0.0258 (10)	0.0196 (10)	0.0151 (9)	0.0046 (8)	0.0057 (8)	0.0052 (8)
C15A	0.0241 (10)	0.0213 (10)	0.0101 (8)	0.0100 (8)	0.0025 (7)	0.0026 (7)
C16A	0.0156 (9)	0.0192 (9)	0.0108 (8)	0.0075 (7)	0.0002 (7)	−0.0021 (7)
C17A	0.0169 (9)	0.0282 (11)	0.0148 (9)	0.0087 (8)	−0.0046 (7)	−0.0052 (8)
C18A	0.0140 (9)	0.0252 (11)	0.0207 (10)	0.0050 (8)	−0.0039 (8)	−0.0090 (8)
C19A	0.0123 (8)	0.0186 (9)	0.0176 (9)	0.0020 (7)	0.0011 (7)	−0.0063 (7)
C20A	0.0162 (9)	0.0193 (10)	0.0267 (11)	−0.0033 (8)	0.0030 (8)	−0.0063 (8)
C21A	0.0220 (10)	0.0189 (10)	0.0274 (11)	−0.0062 (8)	0.0054 (9)	−0.0004 (8)
C22A	0.0193 (9)	0.0191 (9)	0.0158 (9)	−0.0020 (8)	0.0025 (7)	0.0005 (7)
C23A	0.0118 (8)	0.0142 (8)	0.0106 (8)	0.0024 (6)	0.0017 (6)	−0.0028 (7)
C24A	0.0128 (8)	0.0155 (8)	0.0092 (8)	0.0040 (7)	0.0015 (6)	−0.0010 (6)
Zn1B	0.01505 (14)	0.01295 (14)	0.02733 (16)	−0.00137 (8)	0.00488 (10)	−0.00108 (9)
Cl1B	0.0273 (3)	0.0157 (2)	0.0436 (4)	0.00190 (19)	0.0212 (3)	0.0037 (2)
Cl2B	0.0238 (3)	0.0158 (3)	0.0623 (5)	0.00274 (19)	0.0199 (3)	0.0013 (3)
Cl3B	0.0182 (2)	0.0157 (2)	0.0243 (3)	−0.00401 (17)	0.00131 (19)	0.00362 (18)
Cl4B	0.0441 (4)	0.0316 (3)	0.0282 (3)	0.0039 (3)	−0.0148 (3)	−0.0057 (2)
O1W	0.0443 (12)	0.0256 (10)	0.0421 (12)	−0.0001 (8)	0.0005 (10)	−0.0081 (8)

### Geometric parameters (Å, º)

**Table:**

Cr1A—O1A	1.9986 (17)	C8A—H8A	0.9500
Cr1A—N4A	2.0495 (18)	C9A—C10A	1.405 (3)
Cr1A—N3A	2.0619 (17)	C9A—H9A	0.9500
Cr1A—N1A	2.0775 (17)	C10A—H10A	0.9500
Cr1A—N2A	2.0831 (18)	C11A—C12A	1.429 (3)
Cr1A—Cl1A	2.2734 (7)	C13A—C14A	1.401 (3)
O1A—H1O1	0.829 (10)	C13A—H13A	0.9500
O1A—H2O1	0.839 (10)	C14A—C15A	1.380 (3)
N1A—C1A	1.336 (3)	C14A—H14A	0.9500
N1A—C12A	1.362 (3)	C15A—C16A	1.406 (3)
N2A—C10A	1.328 (3)	C15A—H15A	0.9500
N2A—C11A	1.371 (2)	C16A—C24A	1.405 (3)
N3A—C13A	1.336 (3)	C16A—C17A	1.436 (3)
N3A—C24A	1.362 (3)	C17A—C18A	1.353 (4)
N4A—C22A	1.327 (3)	C17A—H17A	0.9500
N4A—C23A	1.366 (3)	C18A—C19A	1.436 (3)
C1A—C2A	1.404 (3)	C18A—H18A	0.9500
C1A—H1A	0.9500	C19A—C23A	1.405 (3)
C2A—C3A	1.372 (3)	C19A—C20A	1.410 (3)
C2A—H2A	0.9500	C20A—C21A	1.378 (3)
C3A—C4A	1.413 (3)	C20A—H20A	0.9500
C3A—H3A	0.9500	C21A—C22A	1.404 (3)
C4A—C12A	1.404 (3)	C21A—H21A	0.9500
C4A—C5A	1.430 (3)	C22A—H22A	0.9500
C5A—C6A	1.361 (3)	C23A—C24A	1.426 (3)
C5A—H5A	0.9500	Zn1B—Cl3B	2.2443 (7)
C6A—C7A	1.432 (3)	Zn1B—Cl2B	2.2744 (8)
C6A—H6A	0.9500	Zn1B—Cl4B	2.2830 (9)
C7A—C11A	1.404 (3)	Zn1B—Cl1B	2.2854 (7)
C7A—C8A	1.414 (3)	O1W—H1OW	0.851 (10)
C8A—C9A	1.376 (3)	O1W—H2OW	0.855 (10)
			
O1A—Cr1A—N4A	174.86 (7)	C8A—C9A—H9A	120.1
O1A—Cr1A—N3A	94.66 (7)	C10A—C9A—H9A	120.1
N4A—Cr1A—N3A	80.23 (7)	N2A—C10A—C9A	122.6 (2)
O1A—Cr1A—N1A	91.43 (7)	N2A—C10A—H10A	118.7
N4A—Cr1A—N1A	93.56 (7)	C9A—C10A—H10A	118.7
N3A—Cr1A—N1A	169.79 (7)	N2A—C11A—C7A	122.96 (19)
O1A—Cr1A—N2A	86.75 (7)	N2A—C11A—C12A	116.85 (17)
N4A—Cr1A—N2A	92.95 (7)	C7A—C11A—C12A	120.19 (18)
N3A—Cr1A—N2A	92.39 (7)	N1A—C12A—C4A	122.94 (18)
N1A—Cr1A—N2A	79.76 (7)	N1A—C12A—C11A	117.07 (17)
O1A—Cr1A—Cl1A	89.79 (5)	C4A—C12A—C11A	119.99 (18)
N4A—Cr1A—Cl1A	91.17 (5)	N3A—C13A—C14A	122.3 (2)
N3A—Cr1A—Cl1A	95.39 (5)	N3A—C13A—H13A	118.9
N1A—Cr1A—Cl1A	92.82 (5)	C14A—C13A—H13A	118.9
N2A—Cr1A—Cl1A	171.72 (5)	C15A—C14A—C13A	119.8 (2)
Cr1A—O1A—H1O1	129 (2)	C15A—C14A—H14A	120.1
Cr1A—O1A—H2O1	115 (2)	C13A—C14A—H14A	120.1
H1O1—O1A—H2O1	103 (2)	C14A—C15A—C16A	119.26 (19)
C1A—N1A—C12A	118.63 (17)	C14A—C15A—H15A	120.4
C1A—N1A—Cr1A	128.22 (14)	C16A—C15A—H15A	120.4
C12A—N1A—Cr1A	113.12 (13)	C24A—C16A—C15A	117.26 (19)
C10A—N2A—C11A	118.07 (18)	C24A—C16A—C17A	118.4 (2)
C10A—N2A—Cr1A	129.02 (15)	C15A—C16A—C17A	124.32 (19)
C11A—N2A—Cr1A	112.69 (13)	C18A—C17A—C16A	121.5 (2)
C13A—N3A—C24A	118.07 (17)	C18A—C17A—H17A	119.3
C13A—N3A—Cr1A	128.73 (14)	C16A—C17A—H17A	119.3
C24A—N3A—Cr1A	113.20 (13)	C17A—C18A—C19A	121.0 (2)
C22A—N4A—C23A	118.51 (18)	C17A—C18A—H18A	119.5
C22A—N4A—Cr1A	128.07 (15)	C19A—C18A—H18A	119.5
C23A—N4A—Cr1A	113.30 (14)	C23A—C19A—C20A	117.3 (2)
N1A—C1A—C2A	121.9 (2)	C23A—C19A—C18A	118.5 (2)
N1A—C1A—H1A	119.0	C20A—C19A—C18A	124.1 (2)
C2A—C1A—H1A	119.0	C21A—C20A—C19A	119.5 (2)
C3A—C2A—C1A	119.7 (2)	C21A—C20A—H20A	120.2
C3A—C2A—H2A	120.1	C19A—C20A—H20A	120.2
C1A—C2A—H2A	120.1	C20A—C21A—C22A	119.3 (2)
C2A—C3A—C4A	119.57 (19)	C20A—C21A—H21A	120.4
C2A—C3A—H3A	120.2	C22A—C21A—H21A	120.4
C4A—C3A—H3A	120.2	N4A—C22A—C21A	122.5 (2)
C12A—C4A—C3A	117.17 (19)	N4A—C22A—H22A	118.7
C12A—C4A—C5A	118.95 (19)	C21A—C22A—H22A	118.7
C3A—C4A—C5A	123.88 (19)	N4A—C23A—C19A	122.77 (19)
C6A—C5A—C4A	121.0 (2)	N4A—C23A—C24A	116.80 (17)
C6A—C5A—H5A	119.5	C19A—C23A—C24A	120.39 (18)
C4A—C5A—H5A	119.5	N3A—C24A—C16A	123.38 (19)
C5A—C6A—C7A	121.1 (2)	N3A—C24A—C23A	116.47 (17)
C5A—C6A—H6A	119.5	C16A—C24A—C23A	120.14 (18)
C7A—C6A—H6A	119.5	Cl3B—Zn1B—Cl2B	111.57 (3)
C11A—C7A—C8A	117.5 (2)	Cl3B—Zn1B—Cl4B	107.54 (4)
C11A—C7A—C6A	118.7 (2)	Cl2B—Zn1B—Cl4B	109.89 (4)
C8A—C7A—C6A	123.7 (2)	Cl3B—Zn1B—Cl1B	107.97 (3)
C9A—C8A—C7A	119.0 (2)	Cl2B—Zn1B—Cl1B	109.29 (3)
C9A—C8A—H8A	120.5	Cl4B—Zn1B—Cl1B	110.56 (3)
C7A—C8A—H8A	120.5	H1OW—O1W—H2OW	105 (2)
C8A—C9A—C10A	119.7 (2)		
			
C12A—N1A—C1A—C2A	0.6 (3)	C24A—N3A—C13A—C14A	−0.4 (3)
Cr1A—N1A—C1A—C2A	−177.22 (15)	Cr1A—N3A—C13A—C14A	−179.76 (16)
N1A—C1A—C2A—C3A	1.1 (3)	N3A—C13A—C14A—C15A	0.1 (3)
C1A—C2A—C3A—C4A	−1.4 (3)	C13A—C14A—C15A—C16A	0.5 (3)
C2A—C3A—C4A—C12A	0.0 (3)	C14A—C15A—C16A—C24A	−0.8 (3)
C2A—C3A—C4A—C5A	179.1 (2)	C14A—C15A—C16A—C17A	178.6 (2)
C12A—C4A—C5A—C6A	−2.3 (3)	C24A—C16A—C17A—C18A	1.3 (3)
C3A—C4A—C5A—C6A	178.6 (2)	C15A—C16A—C17A—C18A	−178.1 (2)
C4A—C5A—C6A—C7A	−0.6 (4)	C16A—C17A—C18A—C19A	−1.2 (3)
C5A—C6A—C7A—C11A	2.5 (3)	C17A—C18A—C19A—C23A	−0.1 (3)
C5A—C6A—C7A—C8A	−176.9 (2)	C17A—C18A—C19A—C20A	179.0 (2)
C11A—C7A—C8A—C9A	1.4 (3)	C23A—C19A—C20A—C21A	1.6 (3)
C6A—C7A—C8A—C9A	−179.1 (2)	C18A—C19A—C20A—C21A	−177.5 (2)
C7A—C8A—C9A—C10A	0.2 (4)	C19A—C20A—C21A—C22A	−1.4 (4)
C11A—N2A—C10A—C9A	1.1 (3)	C23A—N4A—C22A—C21A	1.9 (3)
Cr1A—N2A—C10A—C9A	175.09 (18)	Cr1A—N4A—C22A—C21A	177.73 (17)
C8A—C9A—C10A—N2A	−1.5 (4)	C20A—C21A—C22A—N4A	−0.3 (4)
C10A—N2A—C11A—C7A	0.7 (3)	C22A—N4A—C23A—C19A	−1.7 (3)
Cr1A—N2A—C11A—C7A	−174.29 (16)	Cr1A—N4A—C23A—C19A	−178.16 (15)
C10A—N2A—C11A—C12A	−179.22 (19)	C22A—N4A—C23A—C24A	176.20 (18)
Cr1A—N2A—C11A—C12A	5.8 (2)	Cr1A—N4A—C23A—C24A	−0.2 (2)
C8A—C7A—C11A—N2A	−1.9 (3)	C20A—C19A—C23A—N4A	0.0 (3)
C6A—C7A—C11A—N2A	178.62 (19)	C18A—C19A—C23A—N4A	179.15 (19)
C8A—C7A—C11A—C12A	177.99 (19)	C20A—C19A—C23A—C24A	−177.84 (19)
C6A—C7A—C11A—C12A	−1.5 (3)	C18A—C19A—C23A—C24A	1.3 (3)
C1A—N1A—C12A—C4A	−2.0 (3)	C13A—N3A—C24A—C16A	0.0 (3)
Cr1A—N1A—C12A—C4A	176.07 (15)	Cr1A—N3A—C24A—C16A	179.48 (15)
C1A—N1A—C12A—C11A	177.33 (18)	C13A—N3A—C24A—C23A	−178.80 (18)
Cr1A—N1A—C12A—C11A	−4.6 (2)	Cr1A—N3A—C24A—C23A	0.7 (2)
C3A—C4A—C12A—N1A	1.7 (3)	C15A—C16A—C24A—N3A	0.6 (3)
C5A—C4A—C12A—N1A	−177.40 (19)	C17A—C16A—C24A—N3A	−178.90 (19)
C3A—C4A—C12A—C11A	−177.60 (18)	C15A—C16A—C24A—C23A	179.37 (18)
C5A—C4A—C12A—C11A	3.3 (3)	C17A—C16A—C24A—C23A	−0.1 (3)
N2A—C11A—C12A—N1A	−0.9 (3)	N4A—C23A—C24A—N3A	−0.3 (3)
C7A—C11A—C12A—N1A	179.23 (18)	C19A—C23A—C24A—N3A	177.69 (18)
N2A—C11A—C12A—C4A	178.51 (18)	N4A—C23A—C24A—C16A	−179.14 (18)
C7A—C11A—C12A—C4A	−1.4 (3)	C19A—C23A—C24A—C16A	−1.2 (3)

### Hydrogen-bond geometry (Å, º)

**Table:**

*D*—H···*A*	*D*—H	H···*A*	*D*···*A*	*D*—H···*A*
O1*A*—H1*O*1···O1*W*	0.83 (1)	1.72 (1)	2.536 (3)	170 (3)
O1*A*—H2*O*1···Cl1*B*	0.84 (1)	2.19 (1)	3.0208 (19)	168 (3)
O1*W*—H2*OW*···Cl2*B*	0.86 (1)	2.39 (2)	3.172 (3)	153 (3)
O1*W*—H1*OW*···Cl4*B*^i^	0.85 (1)	2.31 (1)	3.155 (2)	170 (3)

Symmetry code: (i) *x*, −*y*+1/2, *z*−1/2.
